# The efficacy of sorafenib against hepatocellular carcinoma is enhanced by 5‐aza‐mediated inhibition of ID1 promoter methylation

**DOI:** 10.1002/2211-5463.13734

**Published:** 2023-11-22

**Authors:** Jing Meng, Shi Li, Zhao‐qing Niu, Zheng‐qiang Bao, Lei‐lei Niu

**Affiliations:** ^1^ Department of Clinical Laboratory The Second Hospital of Shandong University, Shandong University Jinan China; ^2^ Department of Gastroenterology People's Hospital of Weihaiwei Weihai China; ^3^ Cancer Center The Second Hospital of Shandong University, Shandong University Jinan China

**Keywords:** DNA methylation, drug resistance, hepatocellular carcinoma, ID1, sorafenib

## Abstract

Sorafenib resistance greatly restricts its clinical application in patients with hepatocellular carcinoma (HCC). Numerous studies have reported that ID1 exerts a crucial effect in cancer initiation and development. Our previous research revealed an inhibitory role of ID1 in sorafenib resistance. However, the upstream regulatory mechanism of ID1 expression is unclear. Here, we discovered that ID1 expression is negatively correlated with promoter methylation, which is regulated by DNMT3B. Knockdown of DNMT3B significantly inhibited ID1 methylation status and resulted in an increase of ID1 expression. The demethylating agent 5‐aza‐2′‐deoxycytidine (5‐aza) remarkably upregulated ID1 expression. The combination of 5‐aza with sorafenib showed a synergistic effect on the inhibition of cell viability.

## Graphical abstract

In this study, we demonstrated that ID1 expression is regulated by DNMT3B‐mediated promoter methylation. Upregulation of ID1 by transfection of cells with small interfering RNA targeting DNMT3B reduces resistance to sorafenib in HCC cells. Moreover, we demonstrated that combination of sorafenib with 5‐aza may be a promising strategy for overcoming drug resistance.

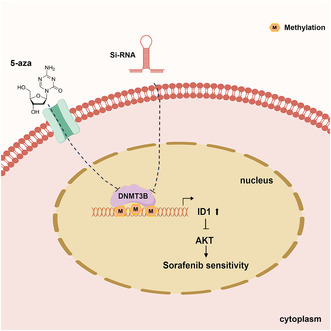

**Abbreviations**
ATF6: activating transcription factor 6
DNMT1: DNA methyltransferase 1
DNMT3A: DNA methyltransferase 3 alpha
DNMT3B: DNA methyltransferase 3 beta
GAPDH: glyceraldehyde‐3‐phosphate dehydrogenase
HCC: hepatocellular carcinoma
ID1: inhibitor of DNA binding 1
MSP: methylmion‐specific PCR
NSCLC: non‐small‐cell lung cancer
PCR: polymerase chain reaction
SASP: senescence‐associated secretory phenotype
STAT3: signal transducer and activator of transcription 3
TCGA: The Cancer Genome Atlas

Although great progress in the understanding of hepatocellular carcinoma (HCC) initiation and development has been made, the incidence and mortality rates of HCC remain high [1]. Due to the rapid tumor growth and weak symptom presentation at early stages, most HCC patients are diagnosed at middle‐late stages and are not eligible for surgical treatments. Sorafenib, a tyrosine kinase inhibitor, is the FDA‐approved first‐line drug for patients with unresectable HCC [2]. Although it exerts survival advantages among HCC patients, the development of drug resistance largely limits its efficacy [3]. Therefore, it is necessary to address resistant mechanisms.

Basic helix–loop–helix (bHLH) transcription factors are ubiquitous regulators of a range of cellular functions. Through heterodimerization and binding to “E box” element of DNA sequences, they control the expression of several genes involved in cell differentiation, proliferation, and survival [4, 5]. The inhibitor of DNA binding/differentiation (ID) family of proteins, which is comprised of four members designated ID1–ID4, possess an HLH domain but lack a DNA binding domain [6]. Thus, they often act as dominant‐negative regulators of bHLH proteins by formating inactive heterodimers with intact bHLH transcription factors [6]. Accumulating evidence proved that ID proteins regulate cell cycle, senescence, or other biological processes [7]. Over the past decades, diverse biological functions of them in cancer have been widely studied. Of them, ID1 is the most investigated. Besides its involvement in tumor malignancy and progression in some cellular contexts [7], more and more researches have been focused on its role in the field of chemotherapy [8]. However, there is a controversial conclusion for the role in drug resistance. Inhibition of ID1 enhanced the effect of temozolomide in glioblastoma, implying that ID1 is a contributor to drug resistance [9]. In contrast, several studies found that ID1 is an inhibitor of chemoresistance. For example, in ovarian cancer, ID1 silencing showed resistance to cisplatin or paclitaxel by inducing the STAT3/ATF6‐mediated autophagy [10]. Ectopic expression of ID1 in non‐small‐cell lung cancer (NSCLC) enhanced cellular sensitivity to epidermal growth factor receptor tyrosine kinase inhibitors through inducing necroptosis by triggering activation of RIP1/RIP3/MLKL pathways [11]. Clinical–statistical analysis demonstrated that surgically treated NSCLC patients with high ID1 expression in primary tumor tissues had better prognoses after adjuvant paclitaxel and cisplatin chemotherapy [12]. In prostate cancer, ID1 mediated docetaxel sensitivity via downregulating p21 [13].

Our previous research proved that ID1 upregulation in HCC is responsible for overcoming sorafenib resistance through inhibiting the p16‐mediated senescence‐associated secretory phenotype (SASP) [14]. We also found that ID1 is differentially expressed in HCC, and the HCC cells with a high expression level of ID1 are more sensitive to sorafenib than those with ID1 low expression [14]. Targeting ID1 is a potent strategy of overcoming sorafenib resistance in HCC patients. Moreover, detecting ID1 expression level would be an effective method of predicting sorafenib sensitivity in clinical, which is helpful to individualized precision therapy. Therefore, it is significant to uncover its regulatory mechanisms in HCC.

5‐aza‐2′‐deoxycytidine (5‐aza) is a typical agent to activate methylated and silenced genes by promoter demethylation. The antitumor effect of 5‐aza in different types of human cancer cell lines, including HCC, has extensively been confirmed [15, 16, 17]. In this study, we found that ID1 expression is controlled by promoter methylation based on the results from methylation‐specific PCR experiment. Functional studies proved that the application of 5‐aza is effective to upregulate ID1 expression and increase the sorafenib sensitivity in HCC cells.

## Materials and methods

### Reagents and antibodies

Sorafenib was purchased from LC Laboratories. 5‐aza and 3‐(4,5‐dimethylthiazol‐2‐yl)‐2,5‐diphenyltetrazolium (MTT) were obtained from Sigma‐Aldrich Chemical Company (Darmstadt, Germany). Primary antibodies to anti‐ID1 and anti‐GAPDH were purchased from Santa Cruz Biotechnology. Antibodies against p‐AKT (ser473), DNMT1, DNMT3A, and DNMT3B were purchased from Cell Signaling Technology (Danvers, MA, USA). Secondary antibodies anti‐rabbit and anti‐mouse IgG conjugated with HRP were purchased from Santa Cruz Biotechnology (Dallas, Texas, USA).

### Cell culture and transfection

The HCC cell lines HepG2, SK‐Hep1, and Hep3B used for this study were purchased from ATCC (Rockville, MD, USA) and stored at liquid nitrogen. All these cells were cultured in DMEM supplemented with 10% heat‐inactivated fetal bovine serum in incubators with a humidified atmosphere of 5% CO2 and 95% air at 37 °C. For RNAi in Hep3B cells, siRNA oligonucleotides targeting DNMT1, DNMT3A, and DNMT3B were ordered from GenePharma, and transfected into cells with Lipofectamine 3000.

### UALCAN and cBioPortal database analysis

UALCAN (http://ualcan.path.uab.edu) database is a comprehensive, user‐friendly, and interactive web resource for analyzing TCGA data [18]. We used it to analyze the relative expression of ID1 in tumor and normal samples. A *t*‐test was used to determine the statistical significance between different levels of ID1 expression. cBioPortal (https://www.cbioportal.org) database is an open platform for providing the visualization, analysis, and download of large‐scale cancer genomics datasets [19]. We downloaded the oncoprint and heatmap of ID1, DNMT1, DNMT3A, and DNMT3B gene expressions in HCC and analyzed the correlations between ID1 and DNMT1, DNMT3A, and DNMT3B, respectively.

### Real‐time quantitative PCR (RT‐qPCR)

Total RNA was extracted using a PureLink® RNA Mini Kit (Invitrogen) according to the manufacturer's instructions. The PCR experiments were performed with SYBR GREEN PCR Master Mix (Takara) in an ABI PRISM 7900 Fast Real‐Time PCR System. All reactions were performed in triplicate for each sample. The primer sequences of ID1 and GAPDH were provided in our previous study [14]. The analysis of relative mRNA expression was carried out using the $2^{-\Delta \Delta C_{t}}$ method, and GAPDH was used as an endogenous housekeeping gene to normalize the mRNA levels.

### Western blot

Cells were washed twice with cold PBS and harvested in RIPA lysis buffer (50 mm Tris–Hcl [pH 7.4], 150 mm NaCl, 1% NP‐40, 2.5% sodium deoxycholate, 1 mm EDTA) in the presence of Protease Inhibitor Cocktail (Pierce, Rockford, IL, USA). Protein concentration was measured using a BCA Protein Assay Kit (Pierce). The expression level of ID1, DNMT1, DNMT3A, and DNMT3B in HCC cell lines was determined by western blot assay. The protein samples were subjected to SDS/PAGE gel electrophoresis, and subsequently transferred to nitrocellulose membrane. Blots were incubated with the indicated antibodies and detected using ECL.

### Cell viability assay

MTT assay was employed to measure cell viability. The details of MTT assay have been described in our previous study [14]. Cells were seeded into 96‐well plates at a density of 1 × 10^3^ cells per well. Each group was analyzed with five to six wells. After incubation cells were treated with different concentrations of sorafenib for 24 h, 10 μL of sterile MTT solution (5 mg·mL^−1^ in PBS) was added into cell supernatant. After 3–4 h, the medium was removed and DMSO (100 μL) was added into each well to dissolve purple formazan crystals. The optical density (OD) was determined at 570 nm using a Bio‐RAD microplate reader (Hercules, CA, USA). Wells without cells served as the blank control. An average of values in each group was calculated after deleting the largest and smallest ones. The experiments were independently triplicated.

### Methylation‐specific PCR (MSP)

Methylation‐specific PCR was carried out according to conventional procedures based on bisulfite‐mediated conversion of unmethylated cytosines to uracil. Genomic DNA (gDNA) was isolated by QIAamp® DNA Mini Kit (Qiagen, Hilden, Germany). 500 ng gDNA was modified with sodium bisulfite using an EZ DNA Methylation™ Kit (Zymo Research, Orange, CA, USA) according to the manufacturer's instructions. Primer MSP‐M (Forward: 5′‐GTAAGGTGATTTTTGTTTAGCGATC‐3′; Reverse: 5′‐ CAAACTAATCTAACTAATTCCCGTA‐3′) was used for methylated reaction, while primer MSP‐U (Forward: 5′‐ GGTAAGGTGATTTTTGTTTAGTGATT‐3′; Reverse: 5′‐CAAACTAATCTAACTAATTCCCATA‐3′) was used for unmethylated reaction. Gray values of the strip images were quantitated utilizing the image j software (Bethesda, MD, USA).

### *In silico* molecular docking of ID1 with 5‐aza

To analyze the binding affinities and modes of interaction between 5‐aza and ID1, AutoDock Vina 1.2.2 (http://autodock.scripps.edu/) (La Jolla, California, USA), a silico protein–ligand docking software was employed. The molecular structure of 5‐aza was retrieved from PubChem Compound (https://pubchem.ncbi.nlm.nih.gov/) [20]. The 3D coordinate of ID1 was downloaded from the SWISS‐MODEL (https://swissmodel.expasy.org/) [21]. For docking analysis, the protein and molecular files were converted into PDBQT format with all water molecules excluded, and polar hydrogen atoms were added. The conformation with the highest score was selected to further analyze. pymol v2.4.1 software (San Carlos, California, USA) was applied for model visualization.

### Luciferase report assay

Deletion mutants of ID1 promoter were kind gifts from Dr. Srikumar Chellappan, who works at the H. Lee Moffitt Cancer Center and Research Institute. The construction method has been concretely described [22]. Hepatocellular carcinoma cells transfected with ID1‐Luc promoter constructs were incubated for 24 h in the presence or absence of 10 μm of sorafenib. Luciferase activity was measured using the dual luciferase assay system (Promega, Madison, WI, USA) according to the manufacturer's protocol. For each construct, relative luciferase activity was defined as the mean value of the firefly luciferase/Renilla luciferase ratios.

### Statistical analysis

Data are shown as mean ± SEM. Statistical differences between the two groups were examined by Student's *t*‐test. *P* values <0.05 were considered statistically significant.

## Results

### The expression of ID1 in HCC is associated with promoter hypermethylation

**Fig. 1:**
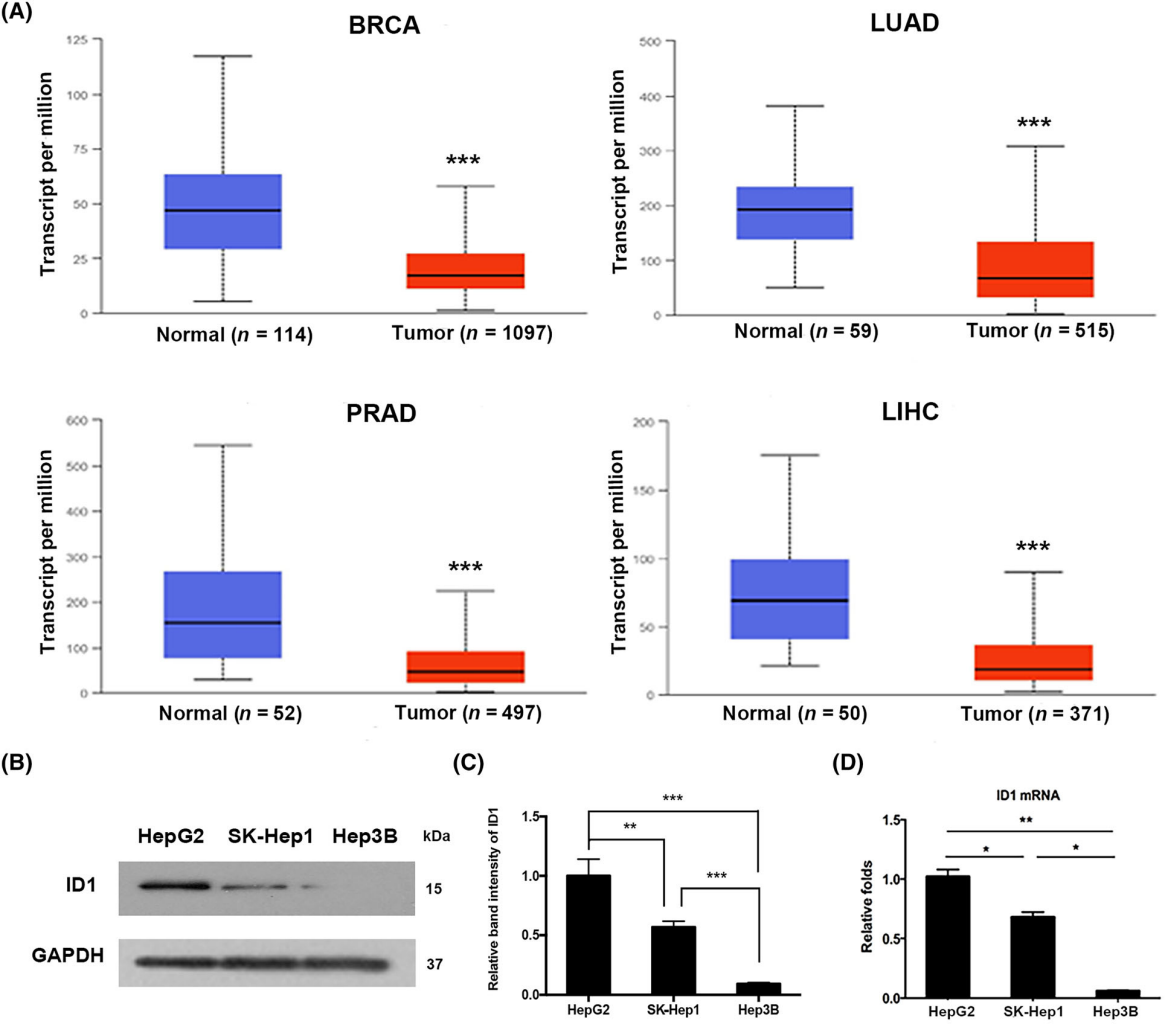
ID1 is downregulated in HCC. (A) The expression levels of ID1 in breast invasive cancer (BRCA), lung adenocarcinoma (LUAD), prostate adenocarcinoma (PRAD), and liver hepatocellular carcinoma (LIHC) were downloaded from UALCAN database. The box‐whisker plots present interquartile ranges (IQRs), including minimum, 1st quartile, median, 3rd quartile, and maximum values. Welch's *t*‐test estimated the significance of differences in expression levels between normal and primary tumors. ***P < 0.001, vs. the Normal group; (B) Western blot experiment was used to observe the different expression levels of ID1 in HepG2, SK‐Hep 1, and Hep3B. (C) The immunoblot band intensities were analyzed by the image j software. The ratio of the ID1 band intensity over the GAPDH band intensity in HepG2 was arbitrarily set at 1.0. The experiments were independently triplicated. Data are shown as mean ± SEM. Statistical differences between the two groups were examined by Student's *t*‐test. **P < 0.01, ***P < 0.001; (D) RT‐qPCR assay was conducted to compare the mRNA levels of ID1 in HepG2, SK‐Hep 1, and Hep3B. All reactions were performed in triplicate for each sample. Data are shown as mean ± SEM. Statistical differences between the two groups were examined by Student's *t*‐test. *P < 0.05, **P < 0.01.

Most of the previous studies reported a high expression of ID1 in many kinds of cancers. It was thought to play crucial roles in cancer development and progression. However, analysis from the TCGA database indicates that ID1 is a generally downregulated gene. As shown in Fig. 1A, the expression of ID1 in some common cancer types, including HCC, breast invasive carcinoma, lung adenocarcinoma, and prostate cancer, is significantly lower in tumor samples than in normal samples. Our previous study revealed that upregulating ID1 expression is beneficial to sorafenib efficacy, it is necessary to elucidate the mechanism of ID1 expression in HCC. Initially, we detected the basal expressions of ID1 among HCC cells. According to the western blot experiment, ID1 was highly expressed in HepG2, moderately expressed in SK‐Hep1, and lowly expressed in Hep3B (Fig. 1B,C). qRT‐PCR results indicated that the expression difference at mRNA levels is consistent with protein levels (Fig. 1D), suggesting ID1 expression in HCC cells is transcriptionally regulated.

**Fig. 2:**
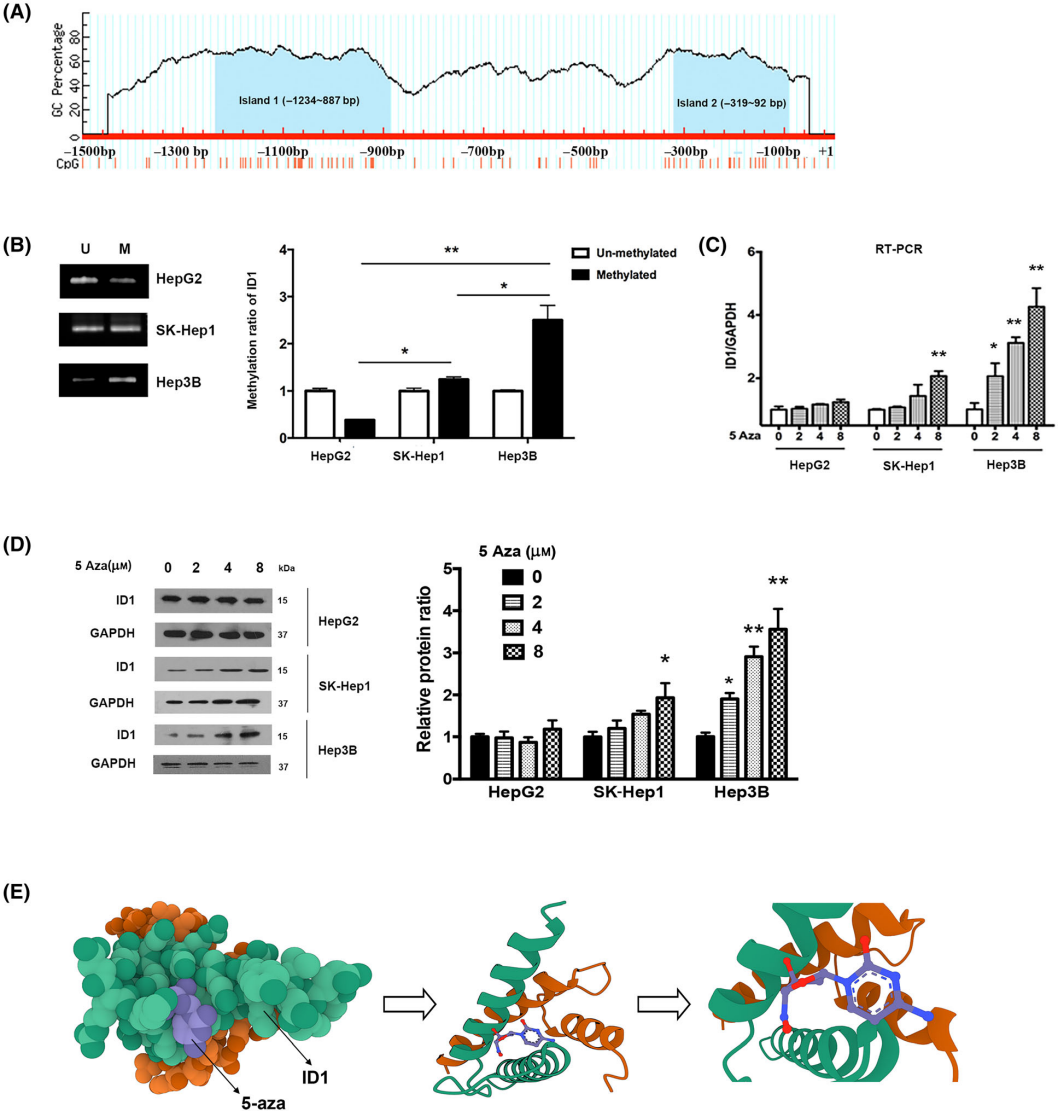
ID1 expression is correlated with promoter methylation. (A) Online software (http://www.urogene.org/methprimer/) prediction of CpG islands in the promoter region (−1500/+1) of ID1. The light blue areas on the map indicate the potential CpG islands. (B) MSP was used to analyze DNA methylation in the ID1 promoter region (left panel), M: Methylation; U: Unmethylation; The experiments were independently triplicated. Band intensity from MSP results was quantified by image j software. The band intensity in un‐methylation group was arbitrarily set at 1.0, data are shown as mean ± SEM. Statistical differences between the two groups were examined by Student's *t*‐test. *P < 0.05, **P < 0.01; (C–D) Effect of 5‐aza on the expression of ID1 at mRNA and protein levels were assessed by RT‐qPCR (C) and western blot (D), respectively. The experiments were independently triplicated, data are shown as mean ± SEM. Statistical differences between the two groups were examined by Student's *t*‐test. *P < 0.05, **P < 0.01, compared with the control group; (E) Three‐dimensional (3D) representation of 5‐aza in complex with ID1 with the highest binding energy of −5.2 kcal·mol^−1^. ID1 dimeric interface is shown in green and orange ribbon. Molecular docking was performed using autodock vina v.1.1.2, and graphics were generated with pymol v2.4.1 software (San Carlos, California, USA).

It has been widely accepted that the methylation of CpG islands in promoter region inhibits gene expression [23]. Using CpG islands prediction software, we identified two typical CpG islands in the ID1 promoter region from −1500 bp to the transcription start site +1 bp (Fig. 2A). To further assess the relationship between the epigenetic regulation and ID1 expression, MSP was performed to evaluate the methylation status of ID1 promoter. As shown in Fig. 2B, ID1 promoter was heavily methylated in Hep3B, moderately methylated in SK‐Hep1, and weakly methylated in HepG2. Together with the data presented in Fig. 1B–D, we speculated that the expression level of ID1 is correlated with promoter methylation.

Next, to further confirm whether the discrepant expression levels of ID1 in different HCC cell lines were due to promoter methylation, we treated the abovementioned three cell lines with a demethylating agent 5‐aza and observed the changes in ID1 expression in both of mRNA and protein levels. We noticed that the expression of ID1 in Hep3B was significantly increased in a dose‐dependent manner. However, only a slight increase in ID1 expression was observed when SK‐Hep1 cells were treated with the highest concentration of 5‐aza. As expected, there was no response for the treatment of 5‐aza in HepG2 (Fig. 2C,D). Taken together, we concluded that ID1 expression is negatively regulated by its promoter methylation. Besides, we performed *in silico* molecular docking analysis to determine the binding affinity of 5‐aza to ID1, data in Fig. 2E showed that 5‐aza exhibited high binding energy of −5.2 kcal·mol^−1^ with ID1, this suggested that 5‐aza had high potential to target ID1.

### DNMT3B is responsible for the CpG methylation of ID1 promoter

**Fig. 3:**
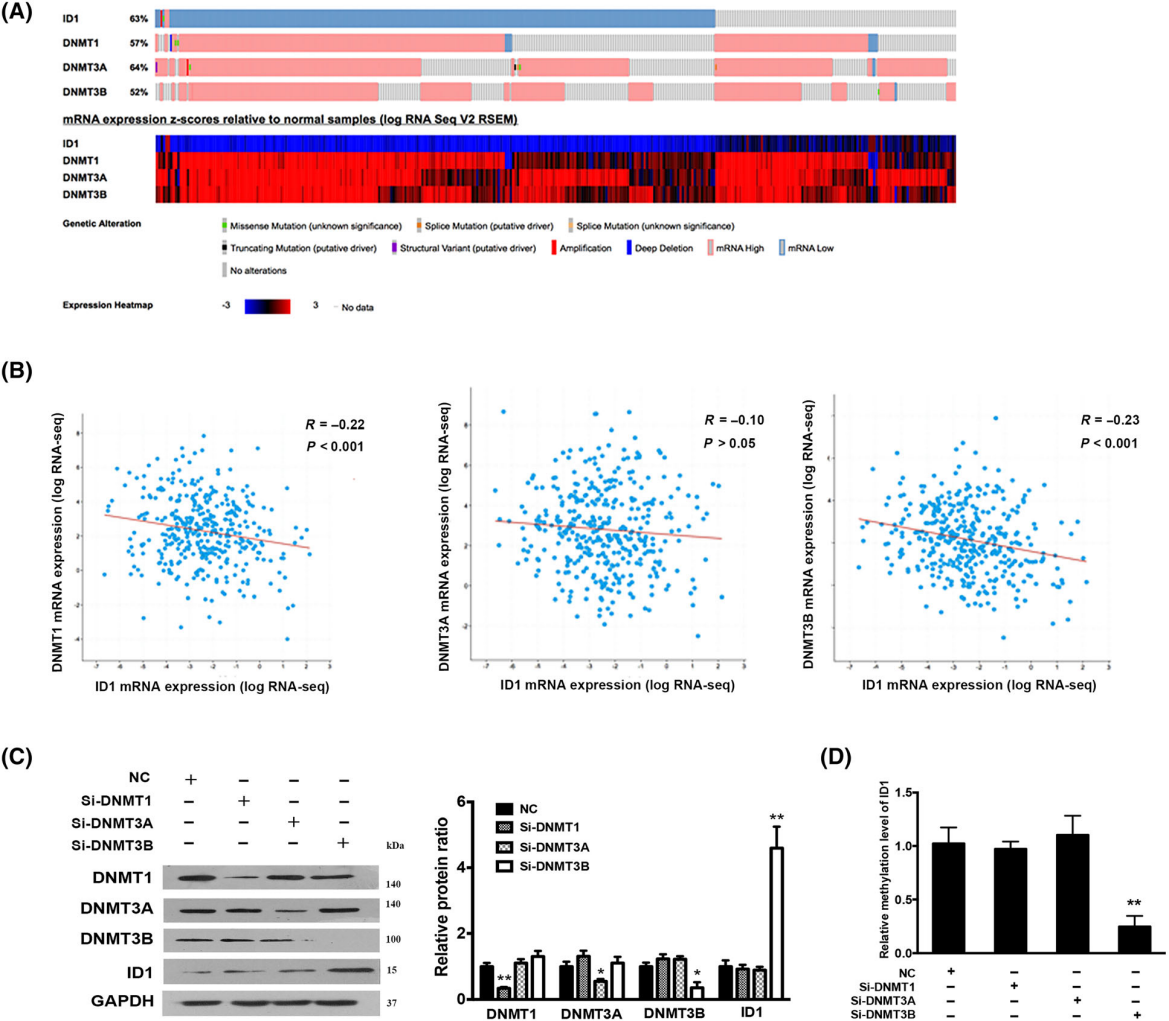
DNMT3B regulates ID1 expression. (A) Oncoporint from cbioportal showing genetic alterations in ID1, DNMT1, DNMT3A, and DNMT3B in HCC from TCGA (*n* = 372) (upper panel); The heatmap shows the mRNA expression levels [*z*‐score normalized log_2_ (FPKM) values] and was generated through cBioPortal (low panel). (B) Correlation of ID1 mRNA expression with DNMT1, DNMT3A, and DNMT3B mRNA expression in HCC were analyzed through cBioPortal, the regression line (red line) depicts the linear association between the two gene expression levels. (C) The protein expression of ID1, DNMT1, DNMT3A, and DNMT3B in Hep3B cells was analyzed after transfection with small interfering RNA (siRNA) targeting DNMT1, DNMT3A, and DNMT3B, respectively. Representative western blotting bands (left panel) and the corresponding densitometric analysis (right panel) are shown. The ratio of the ID1/DNMT1/DNMT3A/DNMT3B band intensity over the GAPDH band intensity in NC group was arbitrarily set at 1.0. The experiments were independently triplicated, data are shown as mean ± SEM. Statistical differences between the two groups were examined by Student's t‐test. *P < 0.05, **P < 0.01, compared with the NC group. (D) MSP was performed to measure the methylation level of ID1 in Hep3B cells after transfection with siRNA targeting DNMT1, DNMT3A and DNMT3B, respectively. Bar chart showing the relative promoter methylation level of ID1 was significantly lower in the si‐DNMT3B group. The experiments were independently triplicated, data are shown as mean ± SEM. Statistical differences between the two groups were examined by Student's *t*‐test. **P < 0.01, compared with the NC group.

CpG methylation is catalyzed by DNA methyltransferases (DNMTs). DNMT1 is the major enzyme responsible for the maintenance of the DNA methylation pattern. DNMT3A and DNMT3B are involved in *de novo* methylation patterns [24]. We speculated that the DNA hypermethylation of ID1 promoter may be induced by DNMTs. To explore which one is responsible for it, we analyzed the genetic alterations of ID1, DNMT1, DNMT3A, and DNMT3B in 372 HCC samples from TCGA dataset. The oncoprint and cluster heatmap indicated that the majority of cases with lower ID1 expression exhibited a tendency to display higher expression of DNMTs (Fig. 3A). Further analysis showed that there is a weak negative correlation (*R* = −0.23) between ID1 and DNMT3B mRNA expression (Fig. 3B). Knockdown of DNMT3B, but not DNMT1 and DNMT3A, activated ID1 expression in Hep3B cells (Fig. 3C). Accordingly, the methylation level was significantly decreased in Hep3B cells transfected with small interfering RNA oligos targeting DNMT3B (Fig. 3D). These data demonstrated that the hypermethylation of ID1 is regulated by DNMT3B.

### 5‐aza enhances the sensitivity of HCC cells to sorafenib by upregulating ID1

**Fig. 4:**
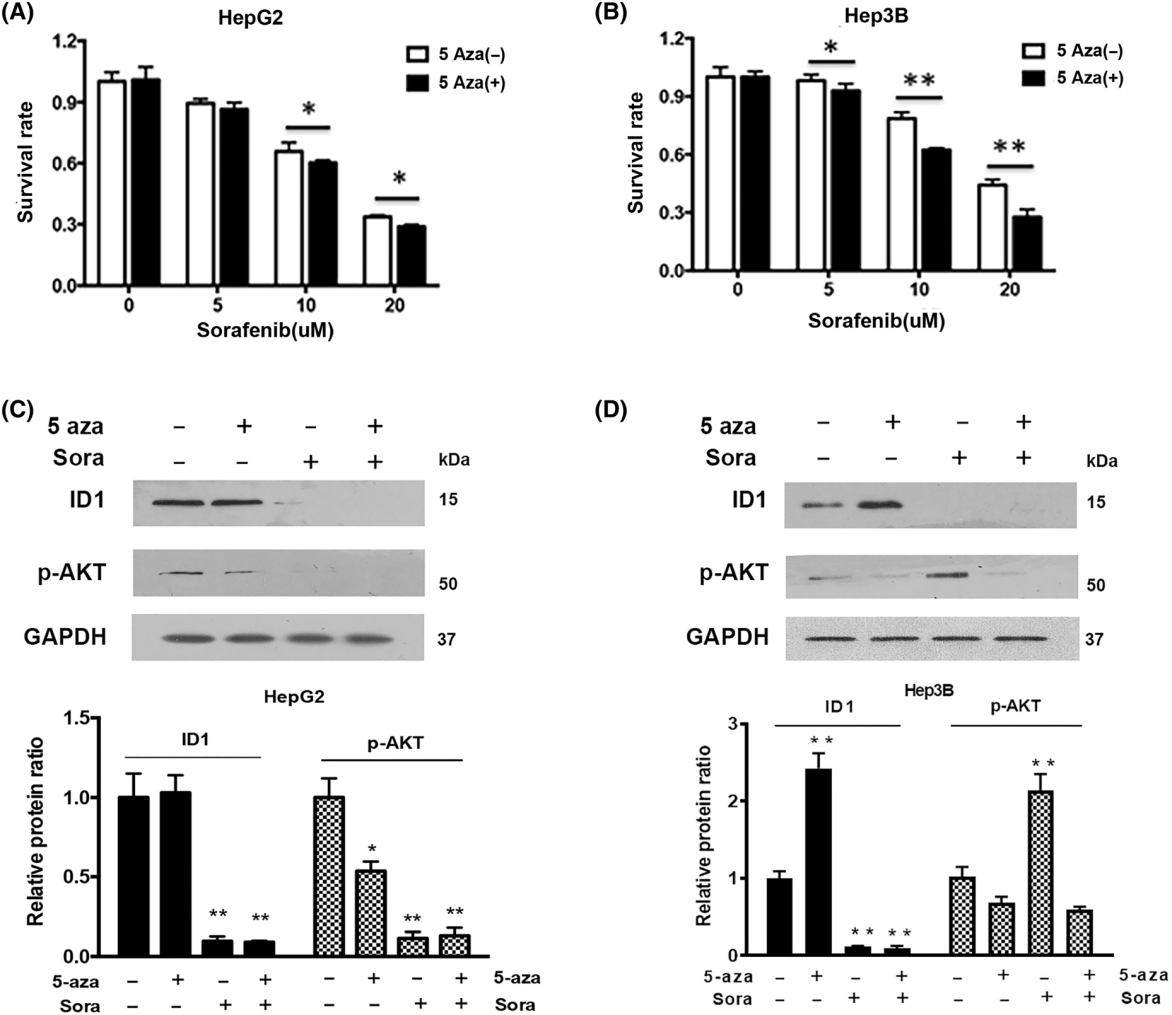
Synergistic effect of 5‐aza on the cytotoxicity of sorafenib in HCC. HepG2 (A) or Hep3B (B) cells were incubated with sorafenib alone or combined with 4 μm of 5‐aza for 24 h. MTT assay was employed to observe the cell viability. The experiments were independently triplicated. Data are shown as mean ± SEM. Statistical differences between the two groups were examined by Student's *t*‐test. *P < 0.05, **P < 0.01, compared with sorafenib alone. The protein levels of ID1 and p‐AKT in HepG2 (C) or Hep3B (D) were detected by western blot. The immunoblot band intensities were analyzed by image j software. The ratio of the ID1 and p‐AKT band intensities over the GAPDH band intensity was arbitrarily set at 1.0. The experiments were independently triplicated, data are shown as mean ± SEM. Statistical differences between the two groups were examined by Student's *t*‐test. *P < 0.05, **P < 0.01.

Our previous study reported that upregulation of ID1 through transfecting overexpression plasmids increases the sensitivity of HCC cells to sorafenib. Considering that 5‐aza was effective to elevate ID1 expression, we would like to know whether it facilitates the antitumor activity of sorafenib. As expected, MTT results showed that the cell viability is much lower in cells treated with 5‐aza combination than sorafenib alone (Fig. 4A,B). Moreover, we noticed that the combination effect is more obvious in Hep3B than in HepG2.

It has been widely accepted that PI3K/AKT signaling pathway plays important roles in tumor survival. Recently, some studies have reported that phosphor(p)‐AKT is activated by sorafenib treatment, which in turn results in the formation of drug resistance [25, 26]. Our research revealed that p‐AKT is the downstream effector of ID1‐involved in the regulation of sorafenib resistance [14]. In this study, the HepG2 cells, in which ID1 is highly expressed, showed more sensitive to sorafenib than Hep3B, we analyzed the changes of ID1 and p‐AKT in the two different HCC cells under the treatment of sorafenib and 5‐aza, by alone or combination. As shown in Fig. 4C,D, 4 μm of 5‐aza remarkably upregulated ID1 expression in Hep3B by up to 2.5‐folds, which was accompanied with a 0.4‐folds decreases of p‐AKT. Sorafenib monotherapy in HepG2 down‐regulated p‐AKT while it was up‐regulated in Hep3B. This result provides an explanation for their different responses to sorafenib. Here, we speculate that the more obvious change for p‐AKT between sorafenib monotherapy and sorafenib‐5‐aza combination therapy in Hep3B than in HepG2 can be used to account for their different response to sorafenib‐5‐aza combination therapy.

### Sorafenib downregulates ID1 expression through inhibiting promoter activity

**Fig. 5:**
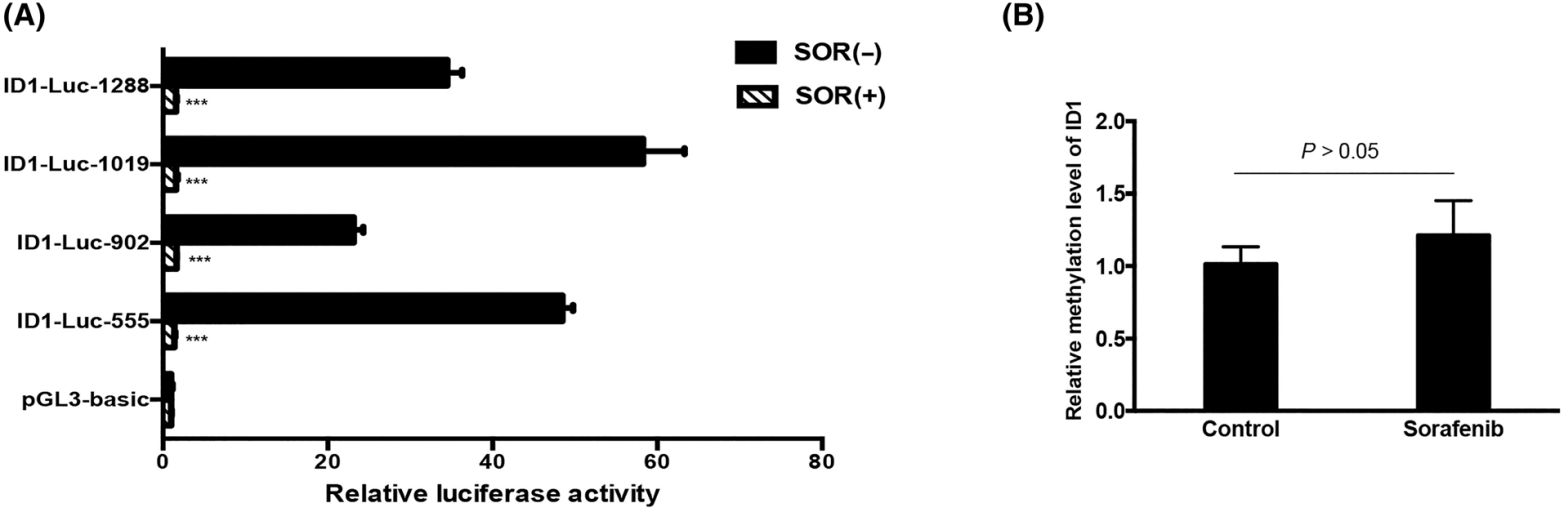
Down‐regulation of sorafenib on the ID1 promoter activity. (A) Continuous truncated ID1 promoter constructs were transfected into cells following sorafenib incubation to determine the relative activity of luciferase. The experiments were independently triplicated, data are shown as mean ± SEM. Statistical differences between the two groups were examined by Student's *t*‐test. ***P < 0.001; (B) Bar chart showing relative promoter methylation level of ID1 in cells with the presence or absence of sorafenib.

We have reported that sorafenib inhibits ID1 expression in a dose‐dependent manner [14]. However, the specific inhibitory mechanism was unknown. In this study, we transfected cells with the different deletion mutants of ID1 promoter to observe the effect of sorafenib on ID1 promoter activity. As shown in Fig. 5A, relative luciferase activity was significantly reduced in the group of cells incubated with sorafenib, which demonstrated that sorafenib downregulates ID1 expression through inhibiting its promoter activity. Next, we conducted an MSP assay to test whether the function of sorafenib‐exerted on promoter activity is relied on methylation site. Consequently, there was no significant change observed for the methylation level of promoter in sorafenib‐treated cells (Fig. 5B). Together with the data from Fig. 5A in which the full length of promoter activity was dramatically silenced, we suggest that there was an indirect mechanism that is responsible for the sorafenib‐induced inhibition of ID1 promoter activity.

## Discussion

Before 2018, sorafenib had been the only FDA‐approved first‐line drug for the treatment of HCC. Although other molecular‐targeted agents, such as lenvatinib, regorafenib, and cabozantinib, showed significant survival benefits and were approved as first‐line or second‐line treatments, sorafenib remains the most commonly used standard of care for these patients [27]. Exploring the resistant mechanism still has important clinical significance.

The role of ID1 in drug resistance is controversial. It is reported to be responsible for chemoresistance in many studies [9, 10]. On the contrary, our and other several findings observed that the ID1 expression is beneficial to chemotherapy [11, 12, 14]. Recent studies showed that senescent cells still have secretory activities, and they may facilitate cancer progress through secreting harmful inflammatory factors into tumor microenvironment, which changes named senescence‐associated secretory phenotype (SASP) [28]. Accumulating evidence proved that components of the SASP, such as IL‐8 or IL‐6, are relative to cancer chemotherapy resistance [29, 30]. In our previous study, we initially found that HCC cells with a high level of SASP showed resistant to sorafenib, further studies elucidated that these cells have a low expression level of ID1 [14]. ID1 overexpression enhanced sorafenib efficacy through downregulating p16 expression, which is an inducer of cell senescence [14]. Therefore, ID1 upregulation is suggested to be a potent strategy for overcoming sorafenib resistance.

Since ID1 expression level is important to sorafenib efficacy, it is reasonable to explore its regulatory mechanisms. Before our research, ID1 had been reported to be a highly expressed gene in HCC. However, comparisons from clinical samples and TCGA database between tumor samples and normal samples indicated that ID1 is lowly expressed, not highly expressed, in HCC. The Cancer Genome Atlas is a public and widely studied cancer database, and it provides extensive reliable RNA sequence value for analyzing differentially expressed genes. A great deal of novel findings has been reported based on database mining. The difference between our and other previous studies is mainly affected by patient population, sample size, and detection methods.

It has been reported that ID1 expression could be induced by 5‐aza in low ID1‐expressing acute myeloid leukemia (AML) cells [31], which resulted in an increase in cell apoptosis; however, this induction could not be explained by direct demethylation of the ID1 gene promoter. In the present study, using CpG islands prediction and MSP experiment, we demonstrated that ID1 is a hypermethylated gene and the methods that inhibiting promoter methylation can be used to restore its expression. We proved that both knockdown of DNMT3B and the application of DNMTs inhibitor 5‐aza in HCC cells upregulated ID1 expression. As an important regulator of DNA methylation, DNMT3B has been proven to be significantly upregulated in HCC tissues and cell lines [32, 33]. Patients with a high level of DNMT3B showed poorer overall survival and shorter metastasis‐free survival [32]. Cellular study indicated that DNMT3B expression was increased in sorafenib‐resistant HCC cells, and DNMT3B‐specific inhibitor nanaomycin A dose‐dependently increased sorafenib sensitivity [34]. Molecular study revealed that this effect is accomplished by the subsequent inhibition of OCT4 [34]. Our present study found that ID1 is a crucial target of DNMT3B, which provides a novel pathway for the DNMT3B‐mediated sorafenib efficacy.

As a well‐characterized demethylating agent, 5‐aza has been identified as a promising chemotherapeutic agent for treating leukemia and melanoma [35, 36, 37, 38]. It enhances chemotherapy sensitivity of various cancer cells [39]. In this study, we discovered for the first time that 5‐aza sensitized HCC cells to sorafenib. Combination of sorafenib with 5‐aza is suggested to be a promising strategy for overcoming drug resistance. Moreover, it can be expected to minimize the sorafenib dose and reduce side effects.

In summary, our findings proved that the low expression of ID1 is correlated with DNMT3B‐mediated promoter methylation. 5‐aza showed a synergistic effect with sorafenib in the sorafenib‐resistant Hep3B cells through upregulating ID1. Targeting DNMT3B/ ID1 pathway would augment the efficacy of sorafenib in HCC.

## Conflict of interest

The authors declare no conflict of interest.

## Author contributions

L‐lN and Z‐qB carried out the experiment. JM, SL, and Z‐qN analyzed and discussed the data. JM and L‐lN wrote the manuscript.

## Data Availability

Data will be made available upon request.
